# RNA demethylase ALKBH5 promotes ovarian carcinogenesis in a simulated tumour microenvironment through stimulating NF‐**κ**B pathway

**DOI:** 10.1111/jcmm.15228

**Published:** 2020-04-24

**Authors:** Yi Jiang, Yicong Wan, Mi Gong, Shulin Zhou, Jiangnan Qiu, Wenjun Cheng

**Affiliations:** ^1^ Department of Gynecology, The First Affiliated Hospital of Nanjing Medical University Nanjing China; ^2^ Department of Gynecology, The Affiliated Huai'an No.1 People's Hospital of Nanjing Medical University Huai'an China

**Keywords:** ALKBH5, ovarian cancer, RNA methylation, tumour microenvironment

## Abstract

Methylation is the main form of RNA modification. N6‐methyladenine (m6A) regulates the splicing and translation of mRNA. Alk B homologue 5 (ALKBH5) participates in the biological regulation of various cancers. However, its role in ovarian carcinogenesis has not been unveiled. In the present study, ALKBH5 showed higher expression in ovarian cancer tissue than in normal ovarian tissue, but lower expression in ovarian cancer cell lines than in normal ovarian cell lines. Interestingly, Toll‐like receptor (TLR4), a molecular functioning in tumour microenvironment (TME), demonstrated the same expression trend. To investigate the effect of abnormal TME on ovarian carcinogenesis, we established an in vitro model in which macrophages and ovarian cancer cells were co‐cultured. In the ovarian cancer cells co‐cultured with M2 macrophages, the expression of ALKBH5 and TLR4 increased. We also verified that TLR4 up‐regulated ALKBH5 expression via activating NF‐**κ**B pathway. Depending on transcriptome sequencing, m6A‐Seq and m6A MeRIP, we found that NANOG served as a target in ALKBH5‐mediated m6A modification. NANOG expression increased after mRNA demethylation, consequently enhancing the aggressiveness of ovarian cancer cells. In conclusion, highly expressed TLR4 activated NF‐**κ**B pathway, up‐regulated ALKBH5 expression and increased m6A level and NANOG expression, all contributing to ovarian carcinogenesis. Our study revealed the role of m6A in ovarian carcinogenesis, providing a clue for inventing new target therapy.

## 1 INTRODUCTION

Epithelial ovarian cancer (EOC) is the third commonest and the most deadly female reproductive cancer. In 2018, 22 240 new cases are estimated in America, including 14 070 deaths.^1^ There is a lack of early‐diagnosis methods for this occult disease bearing few typical early symptoms. When clinical symptoms arise, most of the patients (>70%) have already been mired in the late‐phase in which extensive implantation metastases often occur in the abdominal cavity.^2^, ^3^ TP chemotherapy is routinely given to the patients having undergone cytoreductive surgery, but recurrence may show up at post‐surgery two years, with a median survival of 3‐4 years and a 5‐year survival rate of 40%. Thus, understanding the molecular mechanisms of how ovarian cancer develops is essential to advance diagnostic and therapeutic strategies.

Mounting evidence reveals that ovarian carcinogenesis is an interplay among genetics, epigenetics and transcriptomics. Strikingly, epigenetic regulation may change gene expression to facilitate ovarian cancer development.^4^, ^5^, ^6^ RNA methylation is one form of epigenetic regulation. 6‐methyl adenine (m6A) modification is mostly found in RNA of higher organism. New enzymatic technology has helped researchers to find out Alk B homologue 5 (ALKBH5), a strain of enzyme showing high activity in demethylating m6A in mRNAs or nuclear RNAs. In recent years, with the development of enzymatic technology, types of m6A modification‐related enzymes have been discovered, including WTAP, METTL3 and METTL14 complex, all of which can catalyse the formation of m6A.^7^, ^8^, ^9^, ^10^, ^11^, ^12^, ^13^, ^14^, ^15^ At present, little has been unravelled about the mechanism through which ALKBH5 functions in tumour genesis and development.^16^, ^17^ Zhang found that ALKBH regulated FOXM1 expression to maintain the tumorigenic property of brain glioma‐like stem cells.^18^ However, how ALKBH5 expression drives ovarian cancer development remains unanswered.

Constructed with stromal cells (like firbroblasts, immune cells, mesenchymal stem cells), tumour microenvironment (TME) provides an arena for tumour cells to interact with surrounding normal cells.^19^ Among the inflammatory cells infiltrating into TME, tumour‐associated macrophages (TAMs) make the majority (30%‐50%).^20^ According to their activity and biological functions, macrophages are classified into classic activation of macrophages (M1) and alternative activation of macrophage (M2). More and more studies have found that epigenetic modification can build a tumour microenvironment favourable for the growth of tumour cells. It is also found that the normal cells show epigenetic changes once placed in the supernatant of tumour tissues, although it is not possible to determine who initiates this change. The interaction between the two is factual.^21^ To illustrate this interaction, we used a simulated TME to verify the expression of ALKBH5 in ovarian cancer tissue and the related regulatory mechanism.

## 2 MATERIALS AND METHODS

### 2.1 Cells lines

The ovarian cancer cell lines (SKOV3, HEY, HO8910, OVCAR3) and endometrial cancer cell line (Ishikawa) were obtained from the American Type Culture Collection (ATCC). The monocyte‐macrophage THP‐1 was obtained from China Pharmaceutical University. They were cultured in RPMI 1640 medium (GIBCO) supplemented with 10% foetal bovine serum and 1% (vol/vol) penicillin/streptomycin. Cells were maintained in an incubator containing 5% CO_2_ and 20% O_2_. IL‐4, LPS (lipopolysaccharide) and PMA (Phorbol ester) were obtained from Sigma.

### 2.2 Preparation of cytosolic and nuclear tumour cell extracts

Cytosolic and nuclear fractions were prepared as described previously.^22^, ^23^, ^24^ All procedures were performed at 4°C. Briefly, the cells were homogenized in ice‐cold cytosolic lysis buffer (10 × Pre‐Lysis buffer:100 mmol/L HEPES, pH 7.9; 15 mmol/L MgCl_2_, 100 mmol/L KCl, 0.1 MDTT, protease inhibitor mixture) using an Ultra‐Turrax T8 homogenizer, followed by a 15‐minutes incubation on ice. Homogenates were centrifuged (10 000‐11 000 *g*; 20 minutes, 4°C), and the supernatant (cytosolic fraction) was stored at −70°C. The pellet was resuspended in ice‐cold nuclear lysis buffer (Pre‐Extraction buffer: 20 mmol/L HEPES (pH 7.9), 1.5 mmol/L MgCl_2_, 0.42 mol/L NaCl, 0.2 mmol/L EDTA, 25% (v/v) glycerol, 0.1 mol/L DTT, 1.5 µL protease inhibitor mixture; final volume 150 µL extraction buffer) and incubated for 30 minutes at 4°C. Cellular debris was removed by centrifugation (20 000‐21 000 *g* for 5 minutes, 4°C), and the supernatant (nuclear fraction) was stored at −70°C. Protein concentrations were estimated using Bio‐Rad protein assay and the method of Bradford.^25^

### 2.3 Human macrophage activation and differentiation in vitro

Monocytic THP‐1 cell lines in logarithmic phase were seeded (2 × 10^5‐6^ cells/well). The cells were incubated with 320 nmol/L phorbol ester (PMA) for 24 hours to induce macrophage maturation that was detected with CD68 immunofluorescence; to form M1 macrophages, THP‐1 cells were added to LPS at a final concentration of 100 μg/mL and 18 hours after PMA induction. At the 18th hour, IL‐4 at a final concentration of 20 ng/mL was added to produce M2 macrophages.

### 2.4 Western blotting

Normalized cell homogenates were prepared via boiling one quarter of the volume of concentrated Laemmli buffer (100°C, 5 minutes). Cell extracts (10µg) were resolved on a 10% denaturing gel using running buffer (25 mmol/L Tris, 192 mmol/L glycine, 0.1% (w/v) SDS). See Blue markers (Invitrogen and Life Technologies) were used to determine protein size. Proteins were transferred to nitrocellulose membranes (Hybond ECL; Amersham). Blots were blocked, and Ab hybridization was performed according to the manufacturer's protocols. Proteins were visualized using ECL reagent (Amersham). Protein concentration equivalence was probed by amido black staining and β‐actin Western blot.

### 2.5 RNA isolation and quantitative RT‐PCR

Total RNA isolated with TRIzol reagent (Invitrogen) was reversely transcribed using the Tetro cDNA Synthesis Kit (Bioline). RT‐PCR reactions were performed using SYBR Green Master Mix (Thermo Scientific) on the Step One Plus RT‐PCR (Applied Biosystems). The following primers were used for RT‐PCR: ALKBH5 forward (F), 5′‐TCA GCA TCG GAA CCA GCA AAG‐3′; ALKBH5 reverse (R), 5′‐TCC TGA CTG ACC TTC TTG CTC‐3′; FTO forward (F), 5′‐GTT GGA ACA TGG ATA GCC GC‐3′; FTO reverse (R), 5′‐CAA TGC TGT CGG CAC TTT CA‐3′; NANOG forward (F), 5′‐TTT GTG GGC CTG AAG AAA ACT‐3′ and reverse (R), 5′‐AGG GCT GTC CTG AAT AAG CAG‐3′; SOX2 forward (F), 5′‐GCC GAG TGG AAA CTT TTG TCG‐3′ and reverse (R), 5′‐GGC AGC GTG TAC TTA TCC TTC T‐3′. ACTIN was used as the reference gene for normalization. PCR products were assessed by melting curve analysis. Relative mRNA levels of target genes were calculated by the 2^−ΔΔCt^ method.

### 2.6 In vivo experiments

All animal‐related procedures were performed under Institutional Animal Care and Use Committee (IACUC) protocol 05050*** approved by the Institutional Animal Care and Use Committee of Nanjing Medical University. Female athymic BALB/c nude mice (4‐week‐old) were provided (SLAC Laboratory Animal Co. Ltd.). The animals were raised in a pathogen‐free animal laboratory and randomly divided into the control or experimental group (six mice in each group). The mouse was killed when its weight dropped by over 20%. Tumour volume was measured with fine digital calipers and calculated by the following formula: tumour volume = 0.5 × width^2^×length. *t *Test for two‐group independent samples was performed to determine whether the sample size reached a power of 0.8 and a significance of .05. *P* < .05 was considered statistically significant.

### 2.7 m6A‐Seq

Total RNA was extracted from the co‐cultured ovarian cancer cell lines (HO8910 and A2780) and their controls using TRIzol reagent (Ambion). mRNA was further purified using a dynabeads mRNA purification kit (Ambion, catalog no. 61006) and then submitted to further analysis conducted by Kangchen Biological Engineering Co. Fragmented RNA was subjected to m6A immunoprecipitation (m6A IP) using anti‐m6A rabbit polyclonal antibody (Synaptic Systems; catalog No. 202003) followed by RNA‐Seq.

### 2.8 Immunohistochemistry

Formalin‐fixed and paraffin‐embedded sections (4‐µm thick) from mice biopsies were deparaffinized and rehydrated, followed by antigen retrieval using citrate buffer (pH 6.1). Staining was performed using HIF‐1α (BD Biosciences) or ALKBH5 (Novus Biologicals) antibodies and the LSAB+ System HRP kit (DAKO).

### 2.9 Cell proliferation assay

To detect cell viability, the cells transfected with siRNA were seeded into 96‐well plates (3000 cells/well). After siRNA transfection, cell proliferation was maintained for 24, 48, 72 and 96 hours. CCK8 (10µL/well) was added to each well and incubated at 37°C for 2 hours. Absorbance values at 450 nm were detected by the microplate reader (BioTek). All experiments were performed in quadruplicate. For colony formation assay, 400 cells in each well were seeded into a 60‐mm dish for transfection with siRNA. Having been cultured for two weeks, colonies were fixed with methanol and stained by 0.1% crystal violet. The colonies with a diameter of >1 mm were counted. The treatment was separately performed in three wells and in triplicate. Each experiment was independently repeated for three times.

### 2.10 Flow cytometric analysis

Having been transfected for 48 hours, the cells were collected and washed with ice‐cold PBS. After the double staining with FITC‐Annexin V and propidium iodide (PI) was performed, the FITC Annexin V Apoptosis Detection Kit (BD Biosciences) was utilized according to the manufacturer's instructions. The cells were then analysed with a flow cytometry (FACScan; BD Biosciences) equipped with a CellQuest software (BD Biosciences). Next, the cells were classified into viable cells, dead cells, early apoptotic cells and apoptotic cells. Finally, the relative ratio of early apoptotic cells and apoptotic cells was compared with that of control transfected cells in each experiment.

### 2.11 Statistical analysis

Results were expressed as means with SD. Graphs were created with the SigmaPlot for Windows graphic system (version 1.1; Jandel). Data were analysed with the Student t* test. P* < .05 was considered significant.*P < .05, **P < .01, and ***P < .001. Error bars represented SD of the mean if not stated otherwise. A log‐rank test was used for animal survival.

## 3 RESULTS

### 3.1 ALKBH5 expression level was up‐regulated in human ovarian cancer tissue and down‐regulated in ovarian cancer cell lines

**FIGURE 1:**
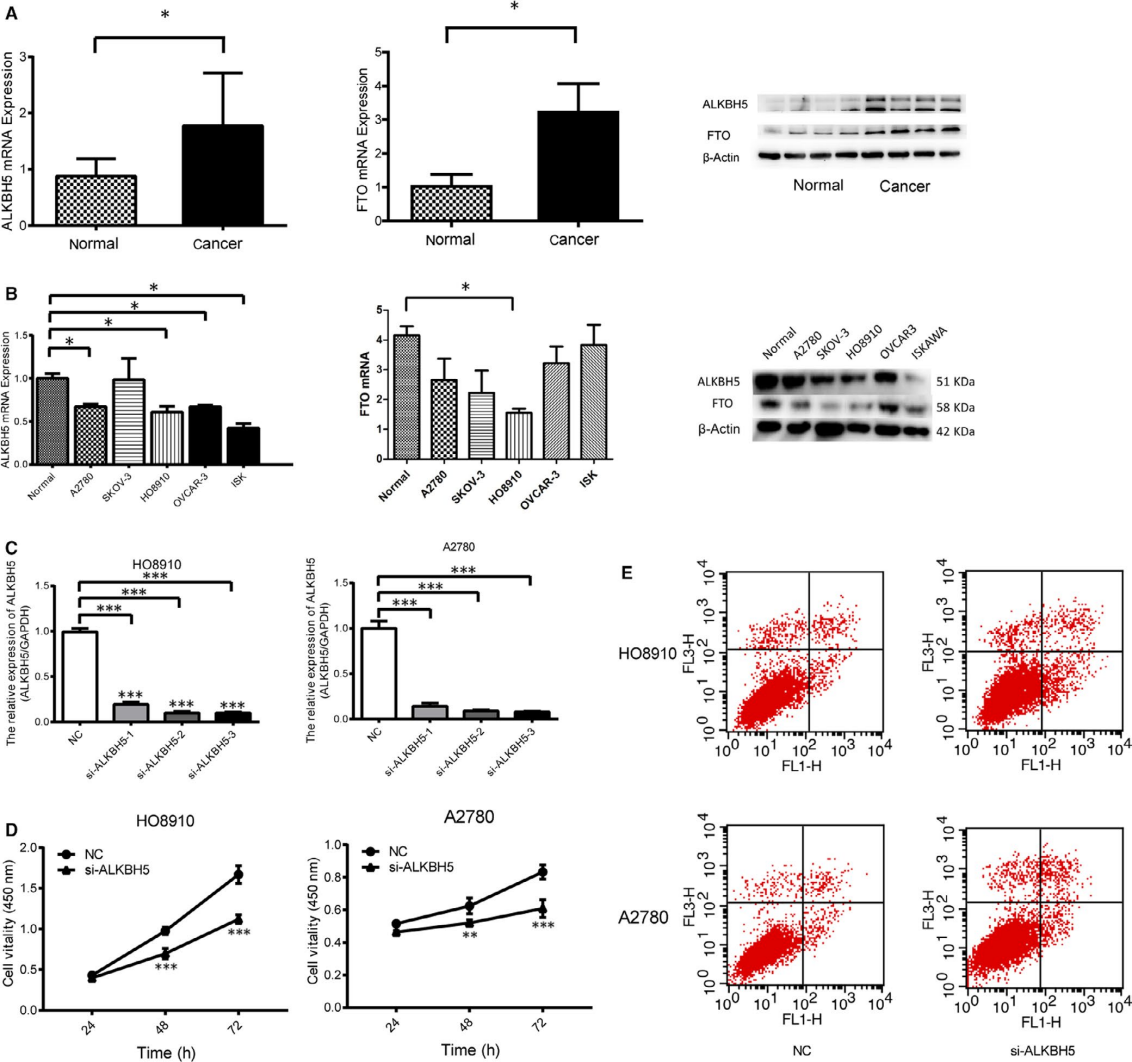
ALKBH5 and FTO expression in ovarian cancer tissues and normal ovarian tissues. A, Relative expression of ALKBH5 and FTO in EOC tissues (n = 73) compared with normal tissues (n = 37). ALKBH5 and FTO mRNA expressions were examined by qPCR and normalized to GAPDH expression, and examined by Western blot which were normalized to β‐actin expression. ALKBH5 and FTO have higher expression in ovarian epithelial cancer tissues than normal ones (*P < .05). B, qRT‐PCR and Western blot analysis of ALKBH5 and FTO mRNA and protein expression in normal ovarian cells (IOSE), EOC cell (A2780, SKOV3, HO‐8910 and OVCAR‐3) and endometrial cancer cell line (Ishikawa). ALKBH5 and FTO have lower expression in ovarian cancer cell lines than normal one (*P < .05). Representative images and data based on three independent experiments. C, Small interfering RNA (siRNA) knocks down the expression of ALKBH5 and detects its interference efficiency separately. The interference sequence si‐ALKBH5 3# has the highest interference efficiency and is selected as the experimental interference sequence. D, Knockdown of ALKBH5 expression could reduce proliferation of A2780 and HO8910 ovarian cancer cells. E, Flow cytometry results show the apoptotic rate was higher in A2780 and HO8910 cells transfected with si‐ALKBH5 than those of controls

We first tested the expression of ALKBH5 in ovarian cancer tissue and cell lines. The results showed that the expression level of ALKBH5 in ovarian cancer tissue was significantly higher than that in normal ovarian tissue (*P* < .05) (Figure 1A). Normal ovarian cell line (IOSE), ovarian cancer cell lines (A2780, SKOV‐3, HO8910 and OVCAR3) and endometrial cancer cell line (Ishikawa) were used to analyse the expression levels of ALKBH5 mRNA. The results showed that the expression level of ALKBH5 mRNA in normal ovarian cell line was significantly higher than those in ovarian cancer cell lines (*P* < .05) (Figure 1B). Furthermore, Western blot found that the protein expression level of ALKBH5 was also increased in ovarian cancer tissue, but decreased in normal ovarian cells (*P* < .05, Figure 1A,B).

### 3.2 Regulatory effects of ALKBH5 on proliferation and apoptosis in ovarian cancer cells

To uncover how ALKBH5 expression regulates the function of ovarian cancer cells, ALKBH5 expression was inhibited by si‐ALKBH5 transfection using Lipofectamine 2000 and the transfection efficacy was tested. Showing higher ALKBH5 expression, two ovarian cancer cell lines A2780 and HO8910 were selected for the following functional experiments (Figure 1C). According to the results, si‐ALKBH53# exhibited the most pronounced transfection efficacy compared with the cells transfected with negative control (NC).

### 3.3 ALKBH5 expression was associated with the proliferation and apoptosis of ovarian cancer cells

To uncover the potential influence of ALKBH5 on the proliferation of ovarian cancer cells, CCK‐8 assay was performed to assess the proliferative ability after knockdown of ALKBH5 in ovarian cancer cells. As shown in Figure 1D, cell number was similar in si‐ALKBH5 group and NC group at 24 hours. At 72 hours, the cell number in si‐ALKBH5 group was significantly lower than that in NC group. The slope of proliferative cell number curve reflected the increasing rate of cell number, that is, the proliferative ability. As a result, knockdown of ALKBH5 significantly decreased the proliferative ability of A2780 cells. Subsequently, Annexin V/PI staining was performed to assess the influence of ALKBH5 on the apoptosis of ovarian cancer cells. As flow cytometry results suggested, the apoptotic rate was higher in A2780 cells transfected with si‐ALKBH5 than those of controls (Figure 1E). Collectively, ALKBH5 could promote the proliferation and inhibit the apoptosis of ovarian cancer cells.

### 3.4 Macrophages were differentiated in simulated TME of ovarian cancer cell lines

To assess whether co‐culture of tumour cells with macrophages can influence ALKBH5 expression, and eventually promote tumorigenesis and invasiveness, we used Transwell model to simulate the local macrophage infiltration microenvironment of human ovarian cancer and explored the role of TAMs in the progression of ovarian cancer. The mononuclear THP‐1 cells were first suspended and dispersed; however, after being treated by PMS for 24 hours, they adhered and clustered, with a pseudopod extending out of each. With flow cytometry, macrophages were found marked by CD68, M1 macrophages by APC‐HLA‐DR and M2 macrophages by PE‐CD163, suggesting the successful induction of differentiation. Ovarian cancer cells were co‐cultured with well‐established M1 and M2 macrophages, respectively. The experiment used (a) ovarian cancer cell lines + TAMs cells (TAM co‐culture group), (b) ovarian cancer cell lines + M1 macrophages (M1 co‐culture group), (c) ovarian cancer cells + M2 macrophages (M2 co‐culture group) and (d) ovarian cancer cell lines only (control group). After 72 hours of co‐culture, the macrophage phenotype highly expressed M2 marker CD163 and lowly expressed M1 marker HLA‐DR, suggesting that the macrophages had already differentiated into M2 phenotypes after the co‐culture with ovarian cancer cells.

### 3.5 ALKBH5 promoted the proliferation and invasion and suppressed the apoptosis of cancer cells in TME

**FIGURE 2:**
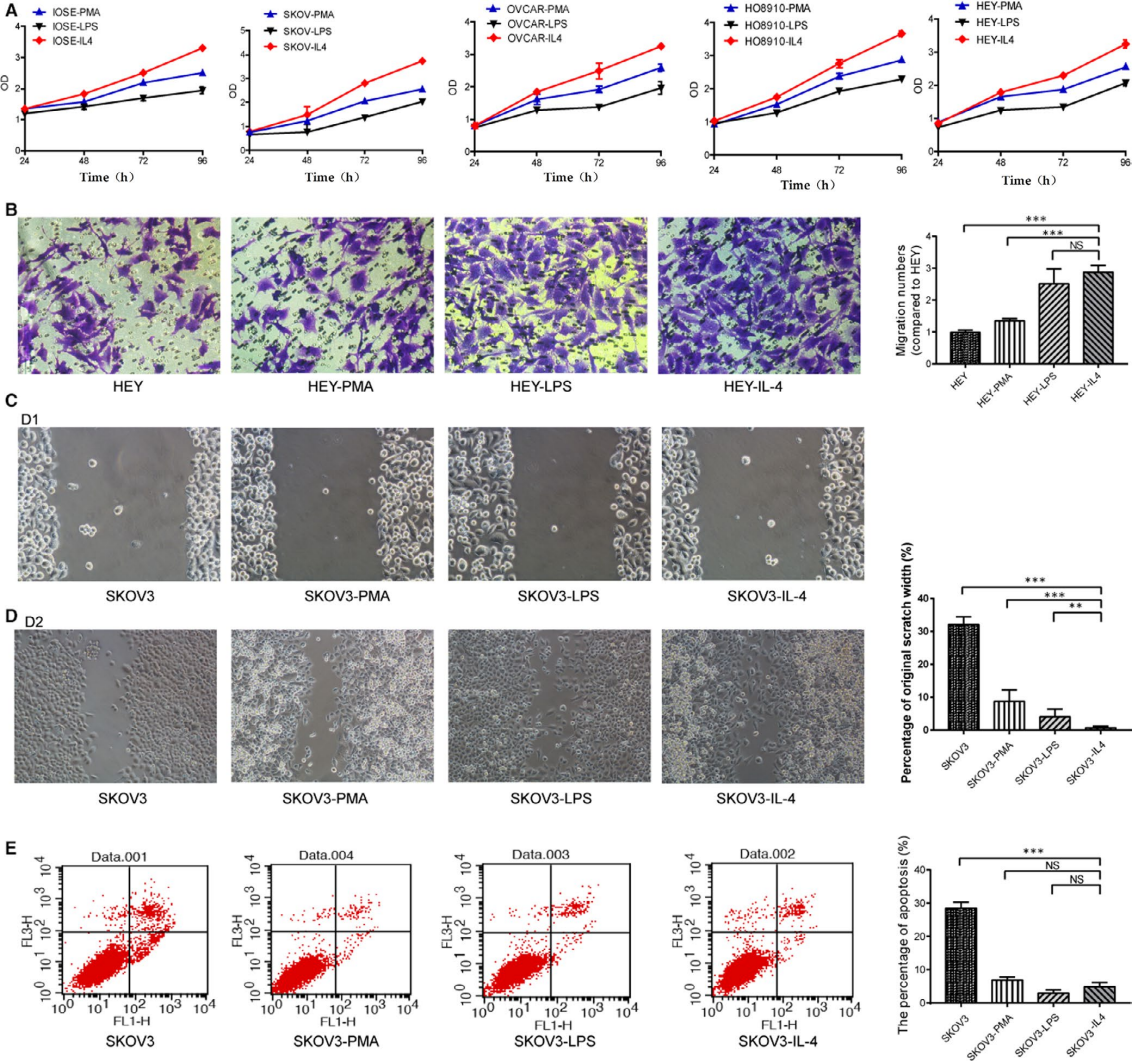
Ovarian cancer cells are co‐cultured with M2 macrophages, the proliferation and invasion ability are enhanced, and the apoptosis ability is decreased. A, Proliferation assessment using CCK8 assay shows that the proliferation rate and activity of ovarian cancer cells in M2 co‐culture group increase, whereas the proliferation rate and activity decrease in M1 co‐culture group. B, Transwell assay shows the increased invasion of the ovarian cancer cells in M2 co‐culture group. One‐way ANOVA indicates differences between groups. C/D, Migration assessment shows in cell wound healing assay, singly cultured cells have decreased migration than cells in M2 co‐cultured group. By comparison, M2 co‐cultured cells have the most significant migration among the groups. E, Flow cytometry Annexin V‐FITC/PI for detection of apoptosis. Ovarian cancer cells (SKOV3, HEY, HO8910, OVCAR3) co‐cultured with M2 macrophages have significantly decreased apoptosis, indicating their increased anti‐apoptotic property compared to the M1 co‐cultured cells and the control

CCK8 assay showed that the proliferative rate of ovarian cancer cells in the M2 co‐culture group increased (Figure 2A), suggesting the enhanced proliferative ability. However, the proliferative rate in M1 co‐culture group slowed down, suggesting the weakened proliferative ability (Figure 2A). Transwell assay showed that the number of HEY cells having migrated through the Matrigel gel and reached the lower layer of the filter was 61.0 ± 6.2 in the HEY control groups and 123.0 ± 8.2 in M2 co‐culture group, respectively (Figure 2B). The cell wound healing assay found that the number of SKOV3 cells in control groups was less than that in M2 co‐culture group, suggesting the enhanced migratory ability of M2 co‐cultured ovarian cancer cells (Figure 2C,D). Then, we used flow cytometry Annexin V‐FITC/PI to detect the apoptosis of ovarian cancer cells. In M2 co‐culture group, the apoptotic ability of SKOV3 cell lines was decreased (Figure 2E).

### 3.6 ALKBH5 significantly promoted ovarian carcinogenesis in vivo

**FIGURE 3:**
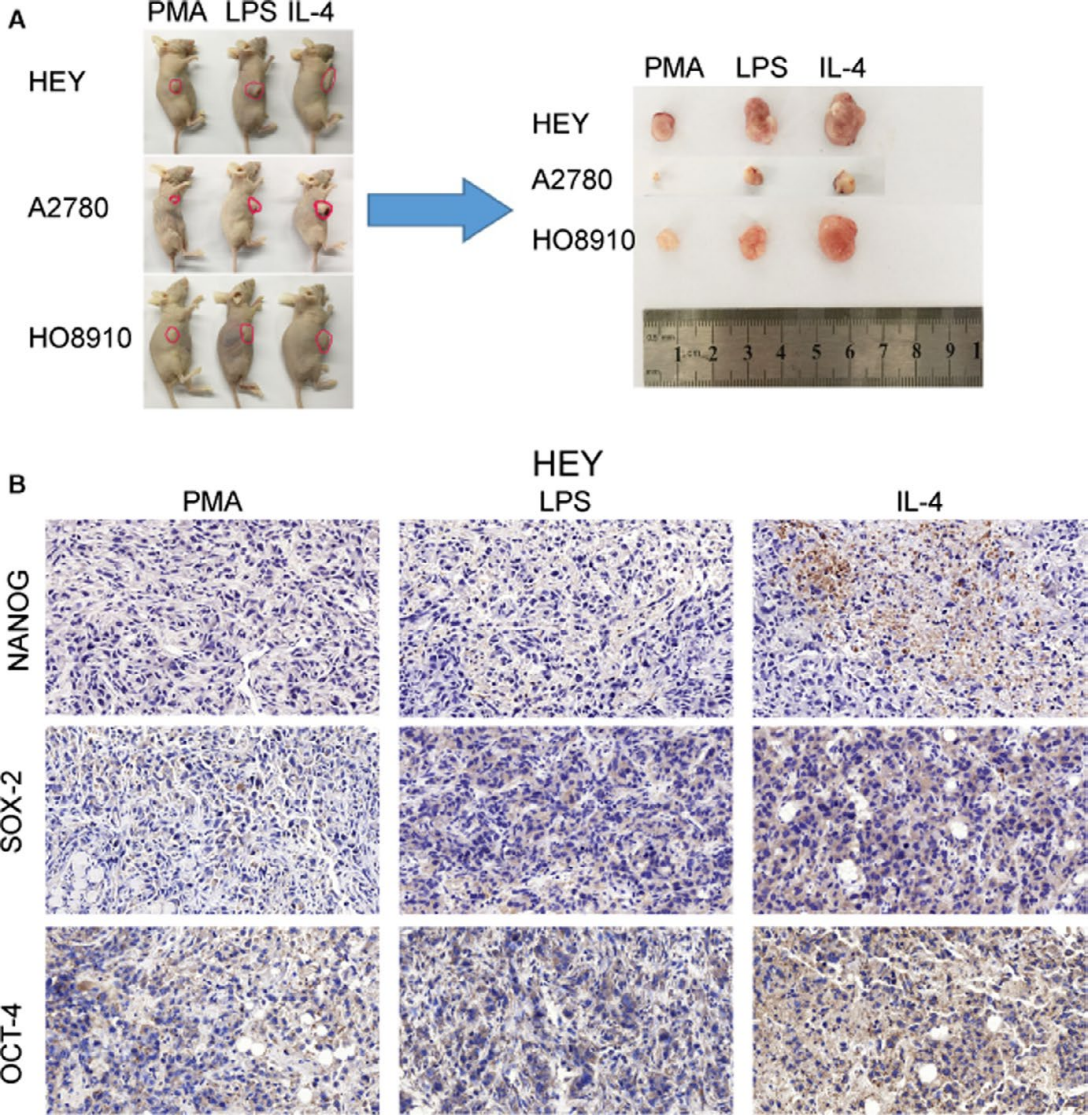
ALKBH5 significantly promoted ovarian carcinogenesis in vivo, after co‐culture of ovarian cancer cells with M2 macrophages. The co‐cultured ovarian cancer cells were subcutaneously inoculated into nude mice (n = 3) in each groups. The tumour volume formed under the skin of ovarian cancer cell lines with M2 co‐cultured group is significantly larger than that of M1 co‐cultured group. The size of tumour formed in the subcutaneous implantation mice model is monitored every 2 days. IHC indicates the overexpression of NANOG, SOX‐2 and OCT‐4 in the nude mice tumours

We further performed subcutaneous injection in nude mice to investigate the effect of ALKBH5 on ovarian cancer growth in vivo. The co‐cultured cell lines were further inoculated subcutaneously into nude mice. The size of subcutaneous xenografts in nude mice was observed every two days. The tumour volume under the skin, where ovarian cancer cell lines co‐cultured with M2 macrophages were inoculated, was significantly larger than that under the skin where ovarian cancer cell lines co‐cultured with M1 macrophage were inoculated (Figure 3). We performed subcutaneous injection experiment in nude mice to test the effect of ALKBH5 on ovarian carcinogenesis. Through this in vivo experiment, we observed that after co‐culture with M1 and M2 macrophages, ovarian cancer cell lines effectively promoted the tumour growth in nude mice, as reflected by the increased tumour size and weight. Taken together, ALKBH5 plays a pivotal role in ovarian cancer growth and metastasis.

### 3.7 ALKBH5 expression level was positively associated with TLR4 expression level, and both genes were differentially expressed in TME with NANOG

**FIGURE 4:**
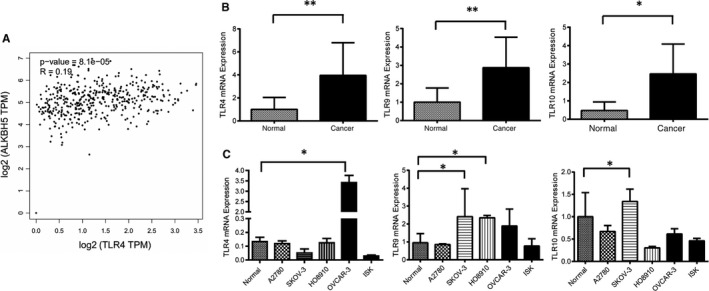
A, The relationship between ALKBH5 and TLR4 was analysed in TCGA data sets. Scatter plot indicated TLR4 and ALKBH5 had positive relationship (R = 0.19). B, Relative expression of TLR4 in EOC tissues (n = 73) compared with normal tissues (n = 37). TLR4 mRNA expression was examined by qPCR and normalized to GAPDH expression. TLR4 has higher expression in ovarian epithelial cancer tissues than normal ones (**P < .01). C, Relative expression of TLR4 in normal ovarian cell line (IOSE) compared with ovarian cancer cell line (OVCAR3). TLR4 mRNA expression was examined by qPCR and normalized to GAPDH expression. TLR4 has higher expression in OVCAR3 cell line than normal one (***P < .00)

Based on the data from TCGA (GSE18520, GSE26193, GSE30161, GSE63885), we found a positive association between TLR4 and ALKBH5 expression (R = 0.19) (Figure 4A).

The expression level of TLR4 was significantly higher in ovarian cancer tissue than in the normal tissue, but lower in ovarian cancer cell lines (*P* < .05) (Figure 4B,C). We found that the expression level of TLR10 in ovarian cancer tissue was significantly higher than that in normal ovarian tissues (Figure 4B). However, the expression level of TLR9 in ovarian cancer cell lines was significantly lower than that in the normal (Figure 4C). Whether these differences were related to the TME change should be answered with more experiments.

**FIGURE 5:**
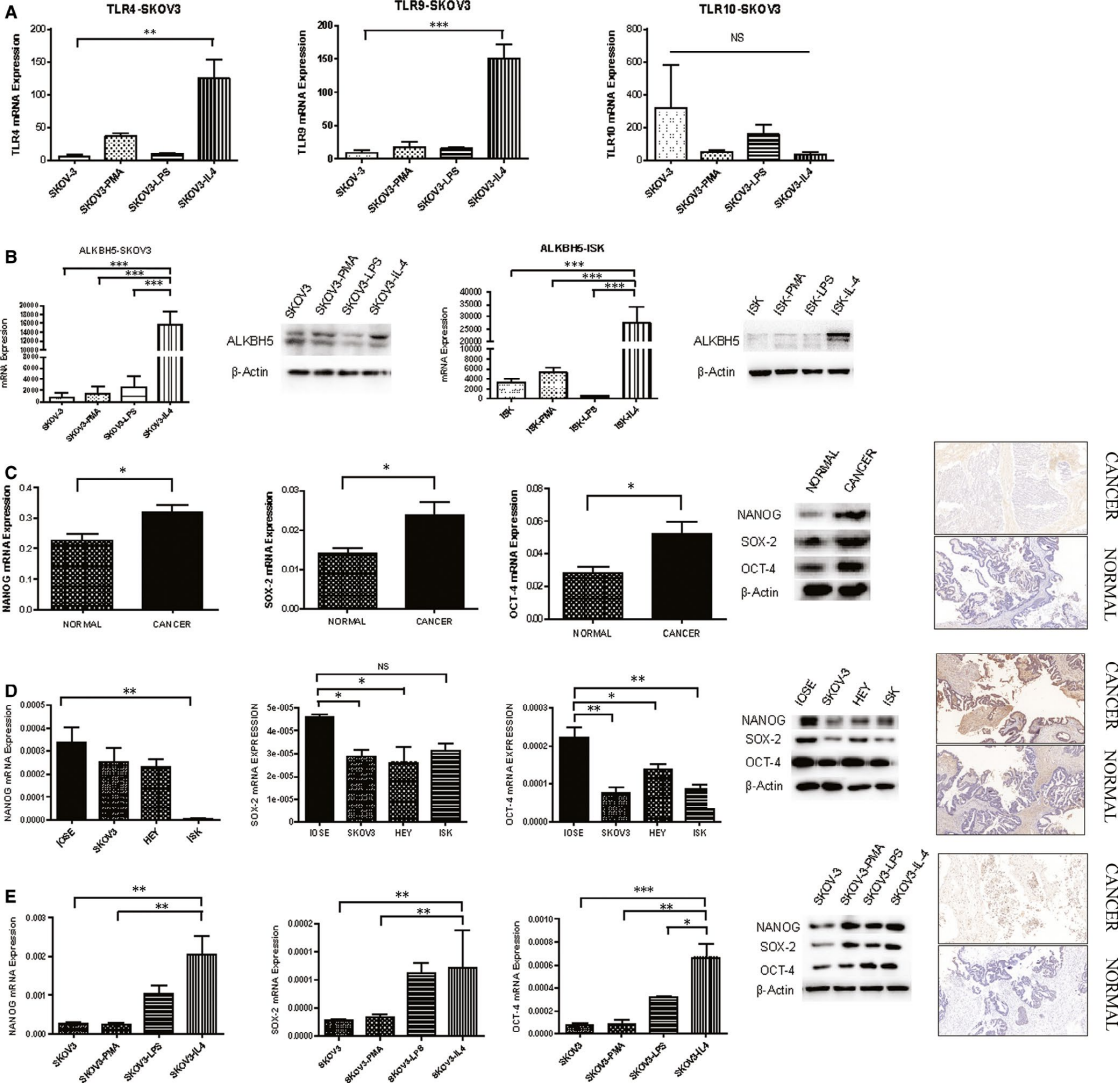
Expression of TLR4, ALKBH5 and NANOG genes after co‐culture of ovarian cancer cells with M2 macrophages. A, The expression of TLR‐related factors in co‐cultured cells. The expression levels of TLR4 and TLR9 in M2‐type co‐cultured cells are significantly higher than those in control cells (***P < .001, **P < .01). B, The expression levels of ALKBH5 significantly increase in SKOV‐3 and Ishikawa M2‐type co‐cultured group cells. ALKBH5 mRNA expression was examined by qPCR and normalized to GAPDH expression, and examined by Western blot which were normalized to β‐actin expression in SKOV3 cell groups. ALKBH5 has higher expression in M2 co‐cultured cells than other groups (***P < .00). C‐D, The expression of NANOG, SOX‐2 and OCT‐4 in normal and malignant ovarian tissues and cell lines. Relative expression of NANOG, SOX‐2 and OCT‐4 in EOC tissues (n = 73) compared with normal tissues (n = 37). NANOG, SOX‐2 and OCT‐4 mRNA expressions were examined by qPCR and normalized to GAPDH expression, and examined by Western blot which were normalized to β‐actin expression. NANOG, SOX‐2 and OCT‐4 have higher expression in ovarian epithelial cancer tissues than normal ones (*P < .05). Relative expression of NANOG, SOX‐22 and OCT‐4 in normal ovarian cell line (IOSE) compared with different ovarian cancer cell lines (SKOV3, HEY) and endometrial cancer cell line (Ishikawa). NANOG, SOX‐2 and OCT‐4 have lower expression in ovarian cancer cell lines than normal one (**P < .01, *P < .05). E, The expression level of NANOG in M2‐type co‐cultured cells was significantly higher than that in other co‐cultured groups (***P < .001, **P < .01, *P < .05)

After detecting the expression of TLR‐related factors in co‐cultured cells, we found that the expression levels of TLR4, TLR9 and TLR10 in M2 co‐cultured cells were significantly higher than those in control cells. The expression levels of TLR4, TLR9 and TLR10 were significantly enhanced in the local macrophage‐infiltrating microenvironment of ovarian cancer (Figure 5A). At the same time, the expression level of ALKBH5 was significantly increased in SKOV‐3 and Ishikawa co‐cultured cells (Figure 5B); meanwhile, all the expression levels of NANOG, SOX‐2 and OCT‐4 increased in ovarian cancer tissues (Figure 5C), but fell in ovarian cancer cell lines (Figure 5D). Having been cultured with M2 cells, the expression levels of NANOG, SOX‐2 and OCT‐4 ascended (Figure 5E).

### 3.8 TLRs regulated ALKBH5 expression through NF‐κB pathway

**FIGURE 6:**
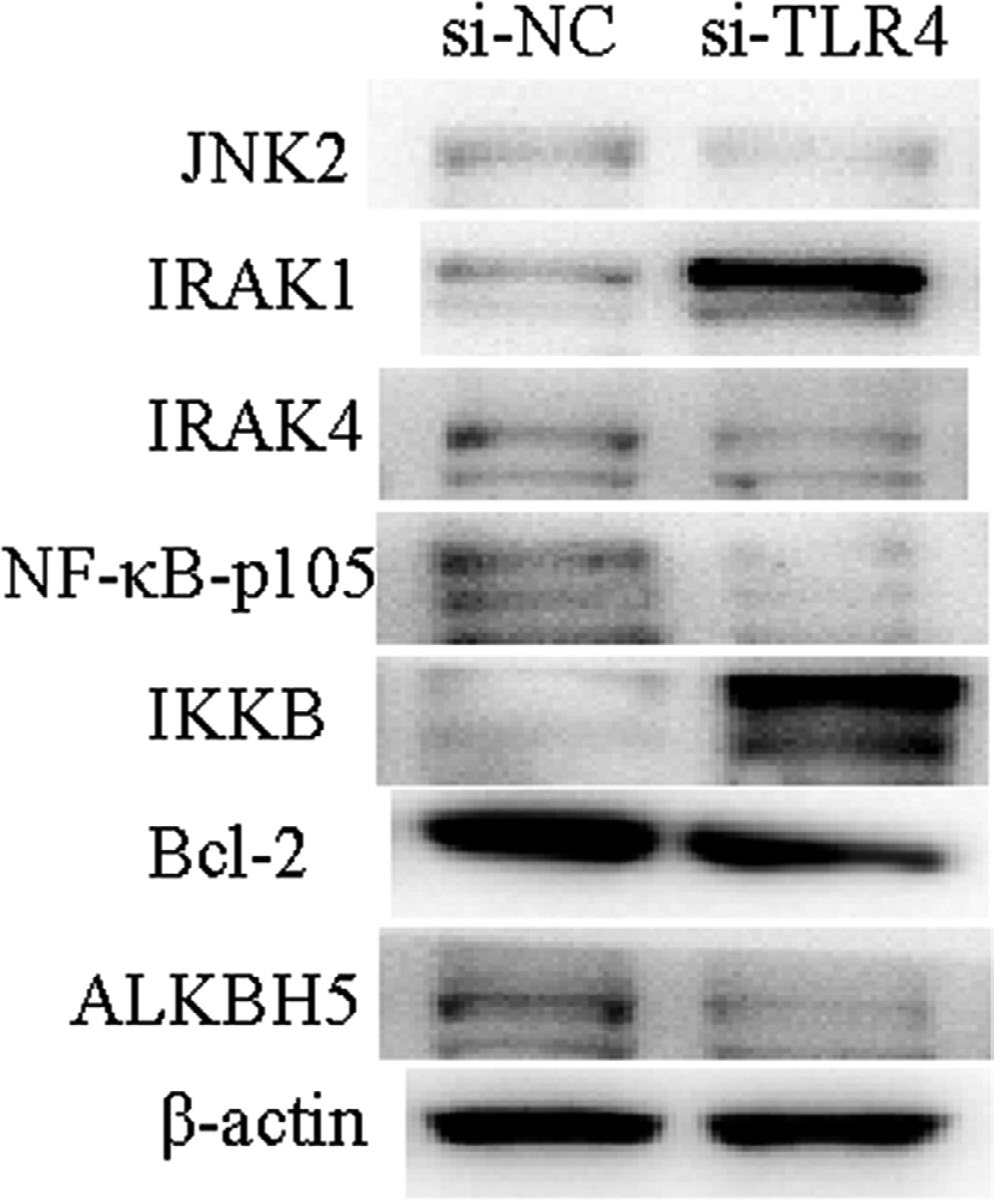
siRNA is used to inhibit the expression of TLR4 in co‐cultured cells. The expression of ALKBH5 decreases; meanwhile, each protein in NF‐**κ**B signalling pathway is detected by Western blot assay. The results show that IRAK1 and IKKB proteins are up‐regulated in si‐TLR4 cells, whereas IRAK4 and NF‐**κ**B‐p105, Bcl‐2 protein expressions decrease

We detected the expression of relevant proteins by Western Blot. The results showed that the protein expression levels of ALKBH5, IRAK1 and IKKB were down‐regulated, and those of IRAK4 and NF‐κB‐p105, Bcl‐2 were up‐regulated in si‐TL4 cells. TLRs positively regulated ALKBH5 expression after the activation of the NF‐κB signalling pathway (Figure 6). Taken together, TLR4 can create an inflammatory microenvironment for ovarian cancer cells through activating NF‐κB signalling pathway.

### 3.9 Expression of ALKBH5 induced the demethylation of NANOG in TME

**FIGURE 7:**
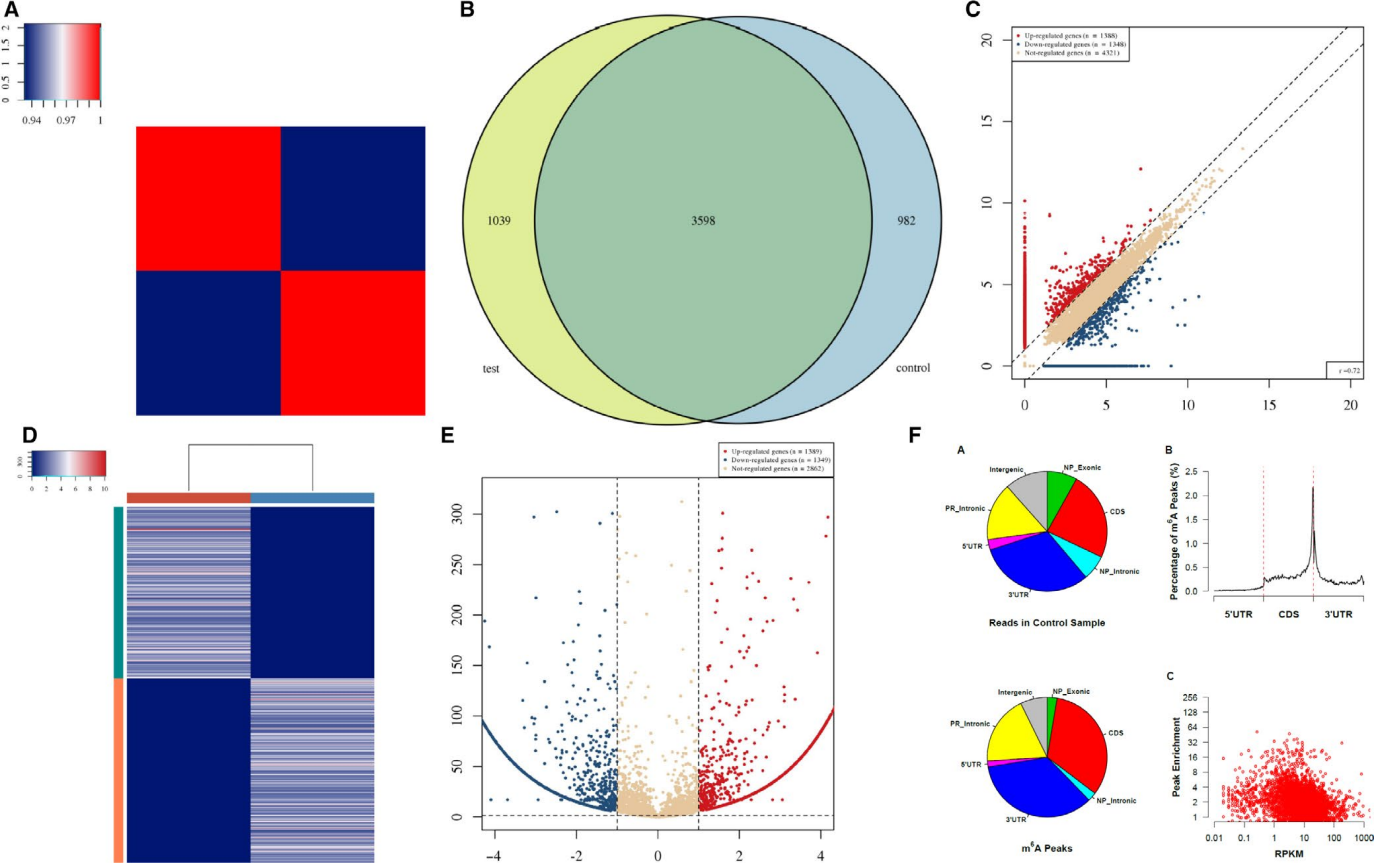
A, Heat map of correlation coefficient from test group and control group. The colours in the panel represent the correlation coefficient of the two samples. Blue shows the two samples have a low correlation coefficient, and red shows the high similarity of the two samples. The plots are performed in R gplots package. B, Venn diagram of the genes number. The plot shows the number of genes methylated in both groups and the number of specific methylated genes. These plots are performed in R VennDiagram package. C, Scatter plot between two groups. RPM values of all identified methylated genes are plotted. The values of X and Y axes in the scatter plot are the averaged RPM values from each group (log2‐scaled). Genes above the top line (red dots, up‐regulation) or below the bottom line (blue dots, down‐regulation) indicate more than 2.0 fold change (default fold change value is 2.0) between the two compared groups. Brown dots indicate methylation level without differentially expression. D, Heat map of gene expression. The heat map shows the 500 genes with the largest coefficient of variation (CV) based on RPM counts. Each row represents one gene, and all the 500 genes are categorized into 10 clusters based on K‐means clustering. Each column represents one sample. The colour in the panel represents the relative expression level (log2‐transformed). The colour scale is shown below: blue represents an expression level below the mean; red represents an expression level above the mean. The coloured bar top at the top panel showed the sample group, and the coloured bar at the right side of the panel indicates the 10 divisions which were performed using K‐means. These plots were performed in R heatmap2 package. E, Volcano plot for test vs control. Red/blue curves indicate 2.0 fold change of differentially methylated gene with statistical significance (red: up‐regulated; blue: down‐regulated). Brown curve indicates non‐differentially methylated gene; fc or *q*‐value is not satisfied. The values of *X* and *Y* axes in the volcano plot are the fold change (log2‐transformed) and *P*‐value (‐log10‐transformed) between the two groups, respectively. F, m6A peak distribution. Transcriptome‐wide distribution of m6A peaks. Pie chart shows the percentage of non‐IP reads (top) and m6A peaks (bottom) within distinct regions of RNA; NP stands for non–protein‐coding genes, whereas PR stands for protein‐coding genes. Distribution of m6A peaks along mRNA (no figure if not all of 5’UTRs, CDSs and 3’UTRs exist). 5’UTRs, CDSs and 3’UTRs of each transcript are separately binned regions spanning 1% of their total lengths; Y‐coordinates represent percentage of m6A peaks located in each bin. Correlation between gene expression level and m6A peak enrichment. The peak enrichment value relative to the transcript abundance within the input RNA is plotted

We analysed the effect of tumour environment on the expression of NANOG/OCT4/SOX2, three core pluripotency factors in human ovarian cancer cells (Figure 5D). In TME, the mRNA levels of NANOG, OCT4 and SOX2 were significantly increased (Figure 5E). To prove that ALKBH5 affects the methylation of NANOG, we performed m6A‐Seq to map the m6A modification in co‐cultured ovarian cancer cells. We identified 3099 m6A‐modified transcripts in SKOV3 co‐culture cells. Pathway analysis revealed that m6A‐modified transcripts in co‐cultured ovarian cancer cell lines were enriched. NANOG was identified as a direct target of m6A modification. In conclusion, NANOG was a downstream target of ALKBH5 that might promote ovarian cancer development through demethanizing NANOG (Figure 7).

## 4 DISCUSSION

In the present study, we demonstrated that the expression of ALKBH5 was elevated in ovarian cancer tissue but lowered in ovarian cancer cell lines. M1 and M2 macrophages were co‐cultured with ovarian cancer cells to simulate a tumour microenvironment, respectively, in which the expression levels of ALKBH5 during transcription and translation were increased. The proliferation, invasion and migration of ovarian cancer cells were enhanced after co‐culture. At the same time, the expression of NANOG, a tumour stem cell factor, increased. It is suggested that the development of ovarian cancer can be regulated by the m6A level of the target genes. In this experiment, expression level of ALKBH5 in ovarian cancer tissue and normal ovarian tissues was first examined. ALKBH5 expression was found to be significantly up‐regulated in ovarian cancer tissue. However, in vitro level of ALKBH5 was lower in ovarian cancer cell lines than that in normal ovarian cell lines.

ALKBH5 is the secondly discovered m6A demethylase following FTO. Dahl et al reported that ALKBH5 is a homologous protein of the human E. coli ALKB dioxygenase family. As a mammalian demethylase, it oxidatively reverses m6A in mRNAs in vitro and in vivo. The demethylation activity of ALKBH5 significantly affects mRNA export, RNA metabolism and assembly of mRNA processing factors in nuclear specks. m6A increases in mRNAs of male ALKBH5‐deficiency mice. The fertility of these mice is impaired due to the apoptosis of spermatocytes during the middle phase of meiosis. Based on this deficiency, Dahl et al identified 1551 differentially expressed genes in mouse testes, covering a wide range of biological functions. These identified genes also interact with spermatogenesis‐related mRNAs in the p53 regulatory network. The discovery of this RNA demethylase strongly proves the basic and extensive functions of reversible m6A modifications in mammalian cells.^26^, ^27^ Different from FTO, ALKBH5 belongs to the ALKB dioxygenase family and has been identified to specifically demethylate m6A in single‐stranded RNAs. Tempel et al reported the crystal structure of ALKBH5 in the presence of its cofactor or ALKBH5 inhibitor citrate. Catalytic assay has demonstrated that the ALKBH5 catalytic domain can demethylate single‐stranded RNA and single‐stranded DNA.^28^ Their results demonstrated that citrate, the TCA cycle intermediate, served as a modest inhibitor of AlkBH5 (IC_50_, ~488 μm). Moreover, m6A binding pocket of ALKBH5 and m6A‐recognized key residues are discovered by mutagenesis and ITC assays. Yang et al proposed that knockout of ALKBH5 triggers nuclear exportation of mRNAs through poly(A)‐tailed RNA FISH, verifying that m6A is involved in this process. The potential role of ALKBH5 in the development of ovarian cancer, however, is rarely reported.^26^

Interestingly, we found that TLR4 and ALKBH5 showed similar expressional trend in simulated TME. We established an in vitro model in which M1 and M2 macrophages were co‐cultured with ovarian cancer cells. In this model, the expression levels of both ALKBH5 and TLR4 increased. TAM has been recognized as a hallmark of nearly all solid malignancies. In EOC, the progression of tumour often correlates with tumour‐associated inflammatory microenvironment,^29^, ^30^, ^31^, ^32^ in which the tumour cells induce the differentiation and polarization of inflammatory cells, and macrophages differentiate into M2 tumor‐associated macrophages. Basic research has shown that TAMs can promote tumorigenesis by shortening the acute phase of inflammation, inhibiting the immune response, promoting tumour cell proliferation and migration, and inducing local immune tolerance. Our results showed that the proliferative rate of ovarian cancer cells in M2 co‐culture group increased, suggesting the enhanced proliferative ability. However, the proliferative rate in M1 co‐culture group slowed down, suggesting the weakened proliferative ability, and that M2 macrophages play a role in promoting ovarian carcinogenesis in TME. The M1 type acts conversely, suggesting that macrophage phenotypes have different effects on the activity of ovarian cancer cells.

The present study convinced that TLR4 up‐regulated ALKBH5 expression through activating NF‐κB signalling pathway. TLR activation by pathogen‐associated molecules such as LPS leads to the release of cytokines and chemokines. These factors create an inflammatory microenvironment after being activated by the NF‐κB signalling pathway.^23^ A few prior investigations demonstrated that high expression of either TLR4 or MyD88 in EOC tissues is associated with the poor survival.^33^, ^34^, ^35^ Inside a tumour are various immune cells, such as macrophages, lymphocytes and dendritic cells, endothelial cells, fibroblasts and perivascular cells. TAMs secret factors such as cytokines and matrix metallo‐proteases (MMPs) that promote the proliferation, invasion and metastasis of tumour cells, as well as tumour vascularization. The data presented in this study verified that tumour cell invasion involves macrophages, a part of the stroma. Co‐culturing macrophages with ovarian cancer cell lines can significantly increase the invasiveness of the tumour cells. Recent studies have demonstrated the role of NF‐κB in malignant progression.^36^, ^37^

Activating NF‐κB in pre‐malignant cells by TNF‐κ and other inflammatory cytokines from the surrounding tumour stroma can transform tumour cells. Indeed, activation of NF‐κB has been found involved in cancerous processes.^38^ By detecting the expression of TLR‐related factors in co‐cultured cells, we showed that the expression levels of TLR4, TLR9 and TLR10 in M2 co‐cultured cells were significantly lower than those in controlled cells. It is suggested that the expression of TLRs, TLR4, TLR9 and TLR10 was significantly reduced in the local macrophage‐infiltrating microenvironment of ovarian cancers. Further research demonstrated that via siRNA‐inhibiting the expression of TLR4 in co‐cultured cells, the expression levels of IRAK1 and IKKB proteins were up‐regulated, and those of IRAK4 and NF‐κB, ‐p105, Bcl‐2 were down‐regulated in si‐TL4 cells, suggesting that TLRs dysregulated ALKBH5 expression and that the NF‐κB signalling pathway was activated. Nevertheless, more studies should be carried to tease out the pathway or pathways directly responsible for carcinogenesis in TME.

Using m6A‐Seq, we also found that NANOG served as a target in ALKBH5‐mediated m6A modification. In the present study, NANOG serves as a critical target gene of ALKBH5. Zhang et al demonstrated that the expression of ALKBH5 was sufficient to mediate a post‐transcriptional induction of NANOG expression and BCSC enrichment.^39^ Zhang also reported that hypoxia induced the HIF‐dependent enrichment of BCSCs in all breast cancer cell lines, which was accompanied by the expressional increase of one or more pluripotency factors (NANOG, KLF4 or SOX2).^40^ As an m6A RNA demethylase, ALKBH5 reduces the m6A levels of NANOG, which in turn leads to the down‐regulation of these two genes at the RNA and protein levels. In the present study, NANOG was just one gene involved in m6A methylation. Its other functions should be exposed with more studies.

The tumour microenvironment is a complex concept, and its role in the development of ovarian cancer needs further clarification. In the microenvironment, which cytokines pass through which signal pathways for certain genes, or by which genes are altered by the methylation status of the genes, is still to be further studied. This study does not delve into the interrelationship between cytokines and gene RNA methylation. In other words, who is the cause and who is the outcome, this needs to be clarified in future research. However, our study uncovered a critical role of mRNA m6A modification in the self‐renewal of ovarian cancer cells, especially in the tumour microenvironment to some extent. This report provides a causative link between mRNA m6A methylation and ovarian carcinogenesis. A therapeutic strategy may be developed to treat ovarian cancer by targeting m6A, its upstream regulators or its downstream targets.

## CONFLICT OF INTEREST

The authors declare that they have no conflict interest in the study or preparation of the manuscript.

## AUTHOR CONTRIBUTIONS

Authors Wenjun Cheng designed the project. Yi Jiang and Yicong Wan contributed to data analysis and prepared the main manuscript. Mi Gong was responsible for part of the article writing and figures editing. Shulin Zhou and Jiangnan Qiu were responsible for clinical data collection and performing clinical validation. Yi Jiang, Yicong Wan and Mi Gong were responsible for proofreading. All authors reviewed the manuscript.

## Data Availability

The data that support the findings of this study are openly available in [PubMed] at https://www.ncbi.nlm.nih.gov/pubmed/, reference number 1, 2, 3, 4, 5, 6, 7, 8, 9, 10, 11, 12, 13, 14, 15, 16, 17, 18, 19, 20, 21, 22, 23, 24, 25, 26, 27, 28, 29, 30, 31, 32.
